# SEOM-SOGUG clinical guideline for localized muscle invasive and advanced bladder cancer (2021)

**DOI:** 10.1007/s12094-022-02815-w

**Published:** 2022-03-26

**Authors:** Begoña P. Valderrama, Aránzazu González-del-Alba, Rafael Morales-Barrera, Ignacio Peláez Fernández, Sergio Vázquez, Cristina Caballero Díaz, Montserrat Domènech, Ovidio Fernández Calvo, Alfonso Gómez de Liaño Lista, José Ángel Arranz Arija

**Affiliations:** 1 Medical Oncology Department, Hospital Universitario Virgen del Rocío, Av. Manuel Siurot, s/n, 41013 Sevilla, Spain; 2 Medical Oncology Department, Hospital Universitario Puerta de Hierro Majadahonda, Madrid, Spain; 3 Medical Oncology Department, Vall d’Hebron Institute of Oncology, Vall d’Hebron University Hospital, Barcelona, Spain; 4 Medical Oncology Department, Hospital Universitario de Cabueñes, Gijón, Asturias Spain; 5 Medical Oncology Department, Hospital Universitario Lucus Augusti, Lugo, Spain; 6 Medical Oncology Department, Hospital General Universitario de Valencia, CIBERONC (Centro de Investigación Biomédica de Red en Cáncer), Valencia, Spain; 7 Medical Oncology Department, Medical Oncology Service, Hospital Fundació Althaia, Manresa, Spain; 8 Medical Oncology Department, Complejo Hospitalario Universitario, Ourense, Spain; 9 Medical Oncology Department, Complejo Hospitalario Universitario Insular-Materno Infantil, Las Palmas, Spain; 10 Medical Oncology Department, Hospital General Universitario Gregorio Marañón, Madrid, Spain

**Keywords:** Bladder cancer, Urothelial, Muscle-invasive

## Abstract

Most muscle-invasive bladder cancer (BC) are urothelial carcinomas (UC) of transitional origin, although histological variants of UC have been recognized. Smoking is the most important risk factor in developed countries, and the basis for prevention. UC harbors high number of genomic aberrations that make possible targeted therapies. Based on molecular features, a consensus classification identified six different MIBC subtypes. Hematuria and irritative bladder symptoms, CT scan, cystoscopy and transurethral resection are the basis for diagnosis. Radical cystectomy with pelvic lymphadenectomy is the standard approach for muscle-invasive BC, although bladder preservation is an option for selected patients who wish to avoid or cannot tolerate surgery. Perioperative cisplatin-based neoadjuvant chemotherapy is recommended for cT2-4aN0M0 tumors, or as adjuvant in patients with pT3/4 and or pN + after radical cystectomy. Follow-up is particularly important after the availability of new salvage therapies. It should be individualized and adapted to the risk of recurrence. Cisplatin–gemcitabine is considered the standard first line for metastatic tumors. Carboplatin should replace cisplatin in cisplatin-ineligible patients. According to the EMA label, pembrolizumab or atezolizumab could be an option in cisplatin-ineligible patients with high PD-L1 expression. For patients whose disease respond or did not progress after first-line platinum chemotherapy, maintenance with avelumab prolongs survival with respect to the best supportive care. Pembrolizumab also increases survival versus vinflunine or taxanes in patients with progression after chemotherapy who have not received avelumab, as well as enfortumab vedotin in those progressing to first-line chemotherapy followed by an antiPDL1/PD1. Erdafitinib may be considered in this setting in patients with FGFR alterations. An early onset of supportive and palliative care is always strongly recommended.

## Introduction and methodology

The highly specialized covering the inner layer of the urinary system from the renal pelvis to the proximal part of the urethra is called transitional cell epithelium or urothelium. Tumors arising from the urothelium are referred as urothelial carcinomas (UC) or transitional cell carcinomas (TCC). Most UC (90%) are located in the bladder, followed by the renal pelvis (8%), ureter, and urethra (2%). UC is four times more common in males than in females and the incidence increases with age and peaks in the seventh and eighth decades of life. Overall, 90% of bladder UC are localized at diagnosis, 75% with the disease confined to the mucosa or submucosa as non-infiltrating or non-muscle-invasive bladder cancer (NMIBC), while 25% infiltrate the detrusor muscle, being muscle-invasive (MIBC). Up to 10% of patients have clinically evident metastases at diagnosis, and approximately one third of patients with localized MIBC will develop metastases after the treatment of the primary tumor [1].

This SEOM-SOGUG guideline has been elaborated with the consensus of ten genitourinary cancer oncologists from Spanish Society of Medical Oncology (SEOM) and Spanish Oncology Genitourinary Group (SOGUG), and it is focused on muscle-invasive and advanced urothelial bladder cancer. Criteria for assigning levels of evidence and grades of recommendation are summarized in Table 1. Statements without grading were deemed justified standard clinical practice by the SEOM/SOGUG faculty and experts. Recommendations are based on current evidence, but the local regulatory status of drugs and procedures should be considered by the reader.

**Table 1:** Levels of evidence/ grades of recommendation

Levels of evidence
(I) Evidence from at least one large randomized, controlled trial of good methodological quality (low potential for bias) or meta-analyses of well-conducted randomized trials without heterogeneity
(II) Small randomized trials or large randomized trials with a suspicion of bias (lower methodological quality) or meta-analyses of such trials or of trials with demonstrated heterogeneity
(III) Prospective cohort studies
(IV) Retrospective cohort studies or case–control studies
(V) Studies without control group, case reports, expert opinions Grades of recommendation
Grades of recommendation
(A) Strong evidence of efficacy with a substantial clinical benefit; strongly recommended
(B) Strong or moderate evidence of efficacy but having limited clinical benefit; generally recommended
(C) Insufficient evidence of efficacy or benefit; does not outweigh risk or disadvantages; optional
(D) Moderate evidence against efficacy or of adverse outcome; generally not recommended
(E) Strong evidence against efficacy or of adverse outcome; never recommended

## Epidemiology and risk factors

Bladder cancer (BC) is the 12th most common cancer type worldwide with an estimated number of 573,278 new cases and 212,536 deaths in 2020. Europe has one of the highest incidence rates of BC in the world, particularly in Southern and Western countries [2]. According to REDECAN, BC will be the 5th most frequently diagnosed in Spain, with 20,613 new cases and 5585 deaths in 2021 [3].

Most bladder tumors have their origin in exposure to factors. Exposure to environmental chemical carcinogens is believed to be responsible for most urothelial tumors including tobacco and occupational aromatic amines, polycyclic hydrocarbons and benzidines. Smoking is the most important risk factor for UC in developed countries. In the NIH-AARP prospective study, the hazard ratio (HR) of incidence and 95% confidence interval (95%CI) was 2.22 in former smokers (95% CI 2.03–2.44;) and 4.06 in current smokers (95% CI 3.66–4.50), and about 50% of the tumors were attributed to tobacco in both sexes. Other well recognized risk factors are schistosomiasis urinary tract infection (endemic in Africa, Asia and South America), pelvic radiation therapy and cumulative doses of cyclophosphamide. Patients with Lynch syndrome have a higher risk of developing urothelial tumors, particularly in the upper tract [4–6].

## Pathological and molecular subtypes of urothelial bladder cancer

### Histology of bladder cancer

Most invasive or advanced bladder tumors (95%) are transitional cell carcinomas (TCC). Some histological variants of UC have been recognized in the 2016 World Health Organization (WHO) classification of tumors of the urinary system, based on morphological features on haematoxylin and eosin–stained pathological sections, such as the UC with divergent differentiation (squamous, glandular, trophoblastic and other rare variations), as well as other morphologic variants (nested, microcystic, micropapillary, lymphoepithelioma-like, sarcomatoid, plasmacytoid, signet ring cell, diffuse, pleomorphic giant cell, lipid-rich, clear cell, and poorly differentiated UC). A 5% of BC are of non-urothelial origin, such as squamous cell (SCC), glandular, urachal, neuroendocrine mesenchymal tumors, and others [7, 8].

### Molecular alterations of urothelial bladder cancer

UC may develop from two molecular pathways via either low-grade papillary or high-grade tumors. Low-grade papillary tumors contain activating FGFR3 mutations, whereas high-grade often contain inactivating mutations in TP53 and/or CDKN2A. Data from the Cancer Genome Atlas Project (TCGA) identified MIBC as one of the tumor types with higher level of genomic aberrations per sample. Tumors were evaluated by DNA copy number, somatic mutation, messenger RNA (mRNA) and microRNA expression, protein and phosphorylated protein expression, DNA methylation, transcript splice variation, gene fusion, viral integration, pathway perturbation, microbe analysis, and the association of clinical and molecular variables with overall survival (OS). Urothelial tumors displayed a large number of somatic DNA alterations, such as 302 exonic mutations, 204 segmental copy number alterations, and 22 rearrangements. There were 64 genes with statistically significant levels of recurrent somatic mutation, such as TP53 (48%), KTDM2D (28%), KDM6A (26%), ARID1A (25%), PI3KCA (22%), RB1 (17%), EP300 (15%), FGFR3 (14%) and TSC1. The TCGA identified five APOBEC mutagenesis signature; the patients with APOBEC-high tumors had improved OS compared to APOBEC-low tumors. Defects in DNA damage repair genes were present in around 30%. They also found 2529 chromosomal rearrangements and structural aberrations. The most common translocation was FGFR3-TACC3 (2%). Most prominent altered pathways were related with cell cycle (93%), receptor tyrosine kinase signaling (72%), chromatin remodeling, and histone-modifying genes (89%), and components of the SWI/SNF nucleosome remodeling complex (64%). Based on these results, around 69% of tumors harbor potentially actionable targets [9–11].

Clustering of mRNA expression levels allowed the identification of 5 molecularly different MIBC subtypes: a) luminal-papillary enriched with FGFR3 alterations; b) luminal-infiltrated characterized by the presence of lymphocytic infiltrates and chemoresistance, and these tumors had increased expression of several immune markers including CD274 (PD-L1) and PDCD1 (PD-1); c) luminal enriched by several uroplakins such as UPK1A, UPK2 and genes associated with differentiated urothelial umbrella cells (KRT20, SNX31); d) basal squamous was characterized by expression of basal and stem-like markers (CD44, KRT5, KRT6, KRT14) and squamous differentiation markers (TGM1, DSC3, PI3) and e) neuronal subtype has mutation in both TP53 and RB1, or TP53 mutation and E2F3 amplification. In an attempt to achieve an international consensus on MIBC using 1,750 MIBC transcriptomic profiles from 16 published datasets cohorts, the authors identified six molecular classes: luminal papillary (24%), luminal no specified (8%), luminal unstable (15%), stroma-rich (15%), basal/squamous (35%), and neuroendocrine-like (3%). The TCGA and consensus attempted to associated molecular classes with clinical implications, prognosis, and therapeutic possibilities. Patients with neuroendocrine-like or neural tumors were associated with the worst prognosis, while patients with luminal papillary subtype were associated with better OS [12, 13].

## Clinical presentation and staging workup

The most frequent form of presentation of BC is macroscopic hematuria followed by irritative symptoms (dysuria, pollakiuria, incontinence and urgency), or obstructive symptoms in locally advanced tumors or those located near the urethra.

The initial diagnostic workup includes physical examination, complete blood count and biochemistry, urinary cytology, upper urinary tract imaging and cystoscopy. The description and localization of all lesions detected at cystoscopy with respect to size, location, number, pattern (solid or papillary) is essential. A fluorescent cystoscopy may increase sensitivity, especially for carcinoma in situ or small papillary lesions. Bimanual examination under anesthesia (EUA) and transurethral resection of the bladder tumor (TURBT) is the method of choice for diagnosis and staging. It is mandatory to include a representation of the muscular layer in the sample obtained. In case of positive cytology and a normal cystoscopy, an upper urinary tract and prostatic urethra exploration should be carried out. Histological diagnosis should be based on the WHO classification [7].

Once diagnosis have been conformed, staging should be completed with a chest, abdomen, and pelvis CT scan, including a nephrographic and excretory urography phase for the study of the upper urinary tract. MRI is an option for selected cases. A bone scan should be performed if there are bone-related symptoms or elevated alkaline phosphatase levels. 18-FDG-PET/CT might help in some cases, but it is not recommended for routine staging of bladder cancer [14]. Staging must be done according to the norms of the American Joint Committee on Cancer staging classification manual 8th edition, 2017 (Table 2) [15].

**Table 2:** TNM staging system for urothelial carcinoma of the bladder

T—Primary Tumour	
Tx	Primary tumour cannot be assessed
T0	No evidence of primary tumour
Ta	Non-invasive papillary carcinoma
Tis	Carcinoma in situ: “flat tumour”
T1	Tumour invades subepithelial connective tissue
T2	Tumour invades muscle
T2a	Tumour invades superficial muscle (inner half)
T2b	Tumour invades deep muscle (outer half)
T3	Tumour invades perivesical tissue:
T3a	microscopically
T3b	macroscopically (extravesical mass)
T4	Tumour invades any of the following: prostate stroma, seminal vesicles, uterus, vagina, pelvic wall, abdominal wall
T4a	Tumour invades prostate stroma, seminal vesicles, uterus, or vagina
T4b	Tumour invades pelvic wall or abdominal wall

N—Regional Lymph Nodes	
Nx	Regional lymph nodes cannot be assessed
N0	No regional lymph node metastasis
N1	Metastasis in a single lymph node in the true pelvis (hypogastric, obturator, external iliac, or presacral)
N2	Metastasis in multiple regional lymph nodes in the true pelvis (hypogastric, obturator, external iliac, or presacral)
N3	Metastasis in a common iliac lymph node(s)

M—Distant Metastasis	
M0	No distant metastasis
M1a	Non-regional lymph nodes
M1b	Other distant metastasis

## Management of locoregional disease

### Radical cystectomy

Radical cystectomy (RC) with regional lymphadenectomy and urinary diversion is the standard surgical treatment of MIBC cT2-T4aN0M0 [16]. In men, RC includes removal of the bladder, distal ureters, and regional lymph nodes as well as the prostate and seminal vesicles, whereas in women, the urethra, uterus, ovaries and part of the vagina should be also resected. Technological developments during the last decade have allowed the implementation of laparoscopic surgery or robot-assisted surgery.

In retrospective studies, the removal of a greater number of lymph nodes was associated with longer survival [17], but the optimal extent of LND has not yet been well established. Standard LND includes removal of the distal common, internal, and external iliac nodes, as well as the obturator and hypogastric nodes. Extended LND, that also includes lymph nodes of the aortic bifurcation, presacral and common iliac vessels, failed to demonstrate a significant advantage in 5-year recurrence-free survival compared with standard LND [18].

### Bladder preservation strategies

Bladder-preserving therapy for MIBC is a reasonable alternative to cystectomy for patients who wish to avoid radical cystectomy and for those who are medically unfit for surgery. If residual disease is present at response evaluation, salvage cystectomy is recommended. Ideal candidates include patients who, after a maximal TURBT, have an unifocal tumor < 5 cm of urothelial histology, absence of carcinoma in situ, clinical stage T2–T3a, no hydronephrosis, and a good bladder function and capacity [19]. Different approaches have been attempted including TURBT alone, TURBT followed by chemotherapy, TURBT followed by radiotherapy, and partial cystectomy. However, none have an established role in MIBC.

A more appropriate strategy seems to be trimodally treatment (TMT), that includes maximal TURBT followed by concurrent chemoradiotherapy (40–45 Gy to the pelvis with concurrent radiosensitizing chemotherapy), and an additional radiation boost to the bladder (20–25 Gy). If residual disease is present at response evaluation, salvage cystectomy is recommended [19]. A multidisciplinary approach including urologists, medical oncologists and radiation oncologists is necessary.

However, no definitive randomized trials have compared bladder-preserving TMT with radical cystectomy. A meta-analysis based upon data from 9000 patients in eight studies found no significant difference in OS, disease-specific survival (DSS), or progression-free survival (PFS) at 5 or 10 years [20]. However, cross-trial comparisons with RC should be avoided due to biases in patient selection and follow-up. In two systematic reviews, this approach obtained 5-year OS rates ranging from 36 to 74%, respectively, with salvage cystectomy rates of approximately 20% in studies with 5-year follow-up [21, 22].

The benefit of adding chemotherapy to RT compared to RT alone is supported by two randomized trials. The first study, that randomized 99 patients to receive radiation with or without cisplatin, demonstrated a statistically significant reduction in the incidence of first pelvic recurrence with the addition of cisplatin (HR 0.50) [23]. In the second trial conducted in 360 patients (BC2001), RT with concurrent mitomycin C and 5-FU improved 2-year locoregional DFS from 54 to 67% (HR 0.68), and 5-year OS from 35 to 48% (HR 0.82), without increasing acute or late toxicity [24]. The optimal chemotherapy regimen has not been defined in adequately powered randomized clinical trials. Alternative regimens are cisplatin plus 5-FU, cisplatin plus paclitaxel and low-dose gemcitabine [25, 26]. Neoadjuvant chemotherapy in TMT has not been shown to improve survival to date [27].

### Perioperative systemic therapies

#### Neoadjuvant treatment

Several randomized trials have explored the benefit of neoadjuvant chemotherapy (NACT). The European Organization for Research and Treatment of Cancer (EORTC) and the Medical Research Council study compared RC versus neoadjuvant CMV (cisplatin/methotrexate/vinblastine). They found an increase in 10-year survival from 30 to 36% and in OS from 37 to 44 months in favor of neoadjuvant chemotherapy [28, 29]. In the Southwest Oncology Group (SWOG) 8710 study, OS was 77 months in patients who received neoadjuvant MVAC (methotrexate, vinblastine, doxorubicin, and cisplatin), compared to 46 months in those who underwent RC alone (HR 1.33). Patients with cT3-T4 tumors had the greatest survival benefit from NACT (65 *vs.* 24 months). In addition, patients receiving MVAC had a significantly higher proportion of pathological complete response (pCR): 38% *vs.* 15% with RC alone. Five-year survival was higher (85%) in the subgroup of patients with pCR. Patients treated with NACT did not have an increased risk of death or surgical complications [30].

Three meta-analyses have also addressed the question of NACT in MIBC. The first included ten trials and found a relative reduction in the risk of death of 9% with NACT vs RC. This benefit was more evident (13%) with platinum-based combinations, that were associated with a 5% absolute 5-year survival benefit [31]. A second meta-analysis with 11 trials (8 of them with cisplatin-based NACT), found an absolute survival benefit of 6% (from 50 to 56%) with NACT, and only 1% chemotherapy-related mortality [32]. The results of the latest meta-analysis also support the use of NACT, with a 5% absolute improvement in survival at five years [33].

There is no evidence for a superior cisplatin-based neoadjuvant regimen. The largest randomized trials used combinations of cisplatin, methotrexate, vinblastine (CMV) or MVAC, but CG (cisplatin/gemcitabine) demonstrated similar pathological pCR rates than those of MVAC, but with a better toxicity profile [34]. Recent results from a randomized trial comparing 6 cycles of dose dense MVAC (ddMVAC) *vs.* 4 cycles of CG in the perioperative setting failed in demonstrating a significant increase in 3-year PFS with ddMVAC, although there was a trend towards a greater benefit with this regimen (3-year PFS: 66% vs 56%, HR = 0.77, 95% IC: 0.57–1.02, p = 0.066). The pCR was 42% with ddMVAC and 36% with CG. ddMVAC was also associated with higher rates of asthenia, anemia, and gastrointestinal side effects [35]. Other cisplatin-free regimens, as carboplatin/gemcitabine, shouldn't be recommended due to their poor pCR rates [36], so that in patients who are ineligible for cisplatin, it is recommended to proceed to surgery rather than giving suboptimal doses of cisplatin or using carboplatin-based regimens.

There is also a lack of evidence on the optimal number of cycles. Most regimens currently administer 4 cycles of NACT, but in the subgroup of patients with pelvic lymph node involvement, which is associated with poor prognosis and it is not sufficiently represented in most studies, 6 cycles can be also recommended [37].

#### Adjuvant treatment

The role of adjuvant chemotherapy (ACT) in high-risk MIBC after RC has been controversial. Three recent randomized trials compared adjuvant chemotherapy with observation. An Italian study included 194 pT2-grade 3 or pT3-T4 patients and examined the effect of adjuvant CG. However, this study was underpowered to demonstrate a benefit in PFS or OS [38]. The second trial, conducted by SOGUG, was closed early because of low accrual, but with 142 patients included, they found a difference in 5-year OS favoring the chemotherapy evaluated CGP (cisplatin/gemcitabine/paclitaxel) arm (60% *vs.* 31%; HR 0.39) [39]. Finally, the EORTC 30,994 study randomized 284 high-risk patients to receive adjuvant chemotherapy either immediately after RC or deferred until relapse. The PFS was longer with the immediate than with the deferred approach (HR: 0.54), but no significant differences in OS were observed [40].

An observational study included 5653 patients with pathological T3-4 or pathological node-positive; 23% of patients received ACT. A stratified analyses adjusted for propensity score demonstrated an improvement in OS with ACT (HR, 0.70; 95%CI, 0.64—0.76), that was consistent in subset analyses, as well as better 5-year OS (37% vs 29.1%) [41].

In an updated metanalysis of ten randomized trials including 1183 individual patient data, cisplatin-based ACT demonstrated a benefit on OS (HR 0.82, 95% CI 0.70–0.96, *p* = 0.02), with an absolute improvement in 5-year OS of 6% (from 50 to 56%), and a 9% absolute benefit when adjusted for age, sex, pT stage, and pN category (HR = 0.77, 95% CI = 0.65–0.92, *p* = 0.004). ACT also significantly improved recurrence-free survival, locoregional recurrence-free survival, and metastasis-free survival with absolute benefits of 11%, 11%, and 8%, respectively, concluding that cisplatin-based ACT is a valid option for improving outcomes in MIBC [42].

Despite the high risk of recurrence, there are no data supporting ACT in patients with pathological evidence of residual disease after NACT, and there are no data with respect to other chemotherapy regimens for patients who are unfit to cisplatin. Checkpoints inhibitors (CPIs) are being evaluating in these situations. A recent phase III trial assigned 709 MIBC patients who refused or were unfit for platinum-based ACT or showed pT2-4a or pN + in the RC specimen after NACT, to receive adjuvant nivolumab or placebo. There was a longer DFS with adjuvant nivolumab in the intention to treat population (median 20.8 months *vs.* 10.8 months; HR 0.70, 95% CI 0.55–0.90) as well as among patients with PD-L1 expression level of 1% or more [43]. OS data is not yet mature. In contrast, a previous reported phase 3 trial with adjuvant atezolizumab did not show a significant difference in DFS or OS [44]. Further results are awaited before considering adjuvant immunotherapy as a standard treatment. Due to lack of evidence, ACT cannot be recommended in patients unable to tolerate cisplatin-based chemotherapy.

## Follow-up strategies for localized bladder cancer

Follow-up should be based on the probability, timing, and most frequent sites of recurrence, as well as on the available salvage strategies. Nearly 90% of local or systemic recurrences of MIBC occur within 36 months after the local treatment of the primary tumor. Local or pelvic recurrence may occur in 5–15% of patients, particularly in those with higher stages, nodal involvement, or positive margins. New urethral UC can be found in 1.5–6% of men with a previous BC, usually after a mean of 13.5–39 months. New upper tract UC (UTUC) occur in 1.8–6.0% of patients and represent the most common site of late recurrence, particularly in patients with multifocal disease, NMIBC with CIS or positive ureteral margins. Diagnosis is based on symptoms in more than a half of patients, urine cytology in 7%, or imaging in 30% of cases. Overall, 50% of patients with MIBC will develop distant metastases, but the percentage is higher in patients with pT3-4 or nodal involvement. Most frequent metastatic sites are lymph nodes, lungs, liver, and bone.

The frequency and duration of follow-up after MIBC should be individualized according to age, comorbidities, and stage of the disease. The EAU guideline recommends a CT scan every 6 months for at least 3 years, and annual imaging thereafter, but closer follow-up may be necessary in high-risk patients, especially after the demonstration of OS increases with the new systemic therapies. CT scans are also recommended for early detection of UTUC. If urethrectomy has not been carried out, cytology by urethral washing should also be performed, as well as cystoscopy and random biopsies in patients with bladder preservation [45, 46].

## Management of advanced/metastatic disease

### First-line systemic treatment and prognostic classifications

Cisplatin-based combination chemotherapy is the standard of care for patients with metastatic disease who are considered candidates (“fit”) for a cisplatin combination therapy, since MVAC was compared with single-agent cisplatin in a prospective randomized trial, demonstrating superiority of MVAC in terms of overall response rate (ORR), PFS and OS (12.5 *vs.* 8.2 months respectively) [47]. Several regimens are considered standard of care in first line: MVAC, ddMVAC and CG. The EORTC conducted a randomized trial that assessed the efficacy of a ddMVAC regimen compared to MVAC regimen. Updated results with a median follow-up of 7.3 years favored ddMVAC in ORR (62% *vs.* 50%, p = 0.06), complete response (CR) rate (21% *vs.* 9%), PFS (median 9.5 *vs.* 8.1 months; HR 0.73, 95% CI 7.0–9.9) and OS (median 15.1 *vs.* 14.9 months, HR 0.76, 95%CI: 0.58–0.099; 5-year OS 21.8% vs 13.5%) [48]. Another phase III trial compared CG with MVAC. OS was considered non-inferior with CG (HR: 1.09; 95%CI, 0.88—1.34), with a median of 14.0 months for CG and 15.2 months for MVAC, and a 5-year OS of 13.0% and 15.3% respectively. Because of lack of significant differences in ORR or PFS, and a better safety profile, CG became the preferred regimen for first-line [49]. Adding taxanes to gemcitabine and platinum showed a trend for improved OS but a higher grade ≥ 3 neurotoxicity [50].

Approximately 50% of patients with advanced BC are considered non-candidates (‘‘unfit’’) for cisplatin-based chemotherapy. Based on an expert consensus, unfitness for cisplatin in clinical trials requires at least one of these five criteria: performance status ≥ 2, creatinine clearance < 60 mL/min, audiometric hearing loss grade ≥ 2, peripheral neuropathy grade ≥ 2, or NYHA class III heart failure [51]. The EORTC 30986 phase II/III trial compared the combination of carboplatin and gemcitabine (CaG) with methotrexate, carboplatin, and vinblastine (M-CAVI) in patients with impaired renal and/or performance score of 2. They found similar OS (median 9.3 and 8.1 months respectively, *p* = 0.64), and lower toxicity with CaG, so it was considered the preferred regimen for unfit patients [52].

To date, CPIs in combination or monotherapy, or their combination with platinum-based chemotherapy have not demonstrated any significant OS benefit over chemotherapy alone in phase III trials. Two phase II, single-arm trials studied the role of immunotherapy in treatment-naive, cisplatin-ineligible patients with locally advanced or metastatic UC. The KEYNOTE-052 evaluated pembrolizumab in 370 patients. The ORR was 28.9% (47.3% in PD-L1 positive tumors), with 9.8% of CR and a median duration of response (DOR) of 33.4 months. Median OS was 11.3 months and 4-year OS was 19% [53]. The IMvigor-210 trial (cohort-1) treated 119 patients with atezolizumab obtaining an ORR of 23% for the entire cohort (28% for IC2/3 PD-L1 positive tumors) and 9% of CR. Median PFS was 2.7 months, median OS was 15.9 months, and 5-year OS was 21.6% [54]. With these results, and other coming from exploratory analyses of some first-line phase III trials comparing CPIs in monotherapy or in combination with chemotherapy *vs.* chemotherapy alone, pembrolizumab and atezolizumab are currently approved by EMA as monotherapy in first-line for patients considered cisplatin ineligible whose tumors express PD-L1 (CPS score ≥ 10% or tumor PD-L1 expression 5% respectively).

Prognostic factors generally reflect tumor biology and the extent of disease and can be used to guide treatment decisions. The presence of visceral metastases (i.e. pulmonary, liver, bone) and a Karnofsky index ≤ 80% were independent negative prognostic factors of poor OS following treatment with first-line MVAC. Median OS for patients with zero, one, or two of these factors were 33, 13.4, and 9.3 months, respectively [55]. These factors have also been validated later for newer regimens.

### Role of immune checkpoint inhibitors and chemotherapy as maintenance or second-line

There are two options for patients with metastatic UC whose disease responds or remains stable after a first-line treatment: switching to a maintenance treatment, or waiting until disease progression. The maintenance strategy was developed after observational studies revealing that only 30–50% receive subsequent lines of systemic therapy. An earlier use of maintenance therapy could increase the number of patients who might potentially benefit from active therapies.

In the JAVELIN Bladder 100 phase 3 trial, 700 patients whose disease did not progress with first-line chemotherapy after four to six cycles of CG or CaG, were randomized to receive best supportive care (BSC) with or without maintenance avelumab until progression of the disease. Maintenance with avelumab significantly prolonged OS in the intention-to-treat (ITT) population as compared with BSC alone. The median OS were 21.4 months in the avelumab group and 14.3 months in the control group (HR: 0.69, 95%CI: 0.56 to 0.86; *p* = 0.001), with 1-year OS of 71.3% and 58.4% respectively. Avelumab also significantly prolonged OS in the PD-L1 positive population: 1-year OS was 79.1% in the avelumab group and 60.4% in the control group (HR 0.56) [56].

Pembrolizumab is the only treatment that has demonstrated benefit in OS as second-line in platinum-refractory patients. The KEYNOTE-045 open-label phase III trial compared pembrolizumab *vs.* standard chemotherapy (paclitaxel, docetaxel, or vinflunine) in 542 patients with advanced UC that had progressed after platinum based chemotherapy. Updated results after more than 5 years of follow-up revealed longer median OS for pembrolizumab compared to chemotherapy (median 10.1 *vs.* 7.2 months; HR 0.71, 95% CI 0.59–0.86), and a 5-year OS of 14.9% *vs.* 8.7% for chemotherapy [57]. The IMvigor-211 phase III trial failed to demonstrate its primary endpoint of an increase in OS with atezolizumab versus chemotherapy in 932 patients with high expression of PD-L1. Due to a hierarchical design, the benefit in other populations could not be formally tested [58]. Other CPIs, as nivolumab or avelumab has showed benefit in small phase I/II trials.

Traditionally, taxanes have been widely used as second line after platinum combinations, although data from randomized trials are lacking. Vinflunine demonstrated an OS benefit in eligible platinum-refractory patients subjects in a phase III study, compared to BSC. The results showed an overall response rate (ORR) of 8.6% and a survival benefit in favor of vinflunine (median OS of 6.9 *vs.* 4.3 month; HR 0.78), with a favorable safety profile [59].

The presence of liver metastases, hemoglobin < 10 g/dl, and ECOG performance status > 0 appear to predict worse outcomes in the second-line therapy for metastatic BC. Four subgroups were identified based on the presence of zero, one, two, or three adverse prognostic factors, with median OS of 14.2, 7.3, 3.8, and 1.7 months, respectively [60]. Additionally, shorter interval from prior chemotherapy appears to be an independent unfavorable prognostic factor [61].

### Treatment options after failure to chemotherapy and immune checkpoint inhibitors

The EV-301 phase III trial compared enfortumab vedotin (EV), an antibody–drug conjugate directed against nectin-4 and linked to an antimicrotubule agent, with standard chemotherapy (paclitaxel, docetaxel, or vinflunine) in advanced UC who has received platinum-containing chemotherapy and progressed during or after treatment with a CPI. OS was longer in the EV arm (12.88 *vs.* 8.97 months; HR 0.70). The ORR was also longer in the EV group (40.6% *vs.* 17.9%). The incidence of AEs were similar in both arms, with rash, peripheral neuropathy and hyperglycemia as events of special interest for EV, that should be closely monitored [62].

Alterations in the fibroblast growth factor receptor (FGFR) gene are common in UC and may be associated with lower sensitivity to CPI treatments. BLC2001 was a phase II study with erdafitinib, a tyrosin kinase inhibitor of FGFR 1–4, in unresectable or metastatic UC with FGFR mutations or FGFR2/3 fusions who had progressed during or following ≥ 1 lines of prior chemotherapy, or were chemotherapy-naïve due to cisplatin ineligibility. A 22% of them had received CPIs after chemotherapy. ORR was 40%, with a median time to response of 1.4 months. The 12 months-PFS and OS were 19% and 55% respectively. AEs of special interest were hyperphosphatemia, ocular events, hand-foot syndrome, dry mouth and skin or nail toxicity [63]. Other antibody–drug conjugates such as sacituzumab govitecan are under investigation in this setting.

## Special issues in non-urothelial bladder carcinomas

Bladder tumors can exhibit UC with divergent differentiation, other morphological variants, and non-urothelial histologies [7]. It should be noted that variant histologies may be under-recognized, although its identification is sometimes relevant for an accurate prognosis estimation and may have therapeutic implications. However, these tumor subtypes are often excluded or underrepresented in clinical trials and therefore, many of the therapeutic recommendations are based in a low level of evidence.

A retrospective dataset from the National Cancer Data Base (NCDB) comprising more than 2000 patients demonstrated an OS gain of NACT before RC in neuroendocrine tumors. Retrospective series show a 44% 5-year survival in patients with small-cell BC (SCBC) treated with chemoradiotherapy [64]. Another retrospective analysis from NCDB including 2187 patients with pT3/4 or LN-positive variant histology disease, failed to demonstrate an OS benefit of adjuvant chemotherapy in patients with non-pure UC, although a numerical trend towards improved survival was seen in neuroendocrine tumors and micropapillary differentiation [65]. Differentiating between urachal and non-urachal subtypes of adenocarcinoma is crucial, since gold standard treatment for localized urachal carcinoma also includes urachectomy and umbilectomy [66] (Table 3).

**Table 3:** SOGUG-SEOM Recommendations for localized muscle-invasive and advanced urothelial bladder cancer

	LE; GoR
Locoregional disease	
Radical cystectomy	
RC with pelvic LND is the standard treatment of MIBC cT2-T4aN0M0	IA
Removal of at least ten lymph nodes is recommended for a correct evaluation of lymph node status	IVA
Bladder preservation strategies	
In experienced centers a TMT bladder-preserving therapy for MIBC is a reasonable alternative to cystectomy for selected patients who wish to avoid or do not tolerate radical cystectomy	IIB
Radiosensitizing regimens as cisplatin or the combination of 5-FU plus mitomycin C are generally recommended	IIB
Other regimens as cisplatin plus 5-FU, cisplatin plus paclitaxel and low-dose gemcitabine are established alternatives	IIB
Other approaches such as TURBT alone, TURBT followed by chemotherapy or TURBT followed by RT are options for patients who cannot tolerate TMT	IIB
Neoadjuvant treatment	
Neoadjuvant cisplatin-based chemotherapy is recommended for patients with T2-4a bladder cancer	IA
Adjuvant treatment	
Adjuvant cisplatin-based chemotherapy is recommended in patients with pT3/4 and or pN + disease after RC if no neoadjuvant chemotherapy has been given and who have no contraindication for cisplatin	IA
Follow-up	
Follow-up after MIBC should be individualized and adapted to the risk of recurrence. Urine cytology and a CT scan should be done every 3–6 months for at least 3 years, and annually thereafter, with urethral washing and cystoscopy in selected cases	VA
Advanced/metastastic disease	
First-line systemic treatment	
Cisplatin-based chemotherapy is considered the standard option for first-line metastatic UC. CG is preferred over MVAC and ddMVAC due to its better safety profile	IA
For unfit patients, GCa should be the preferred first-line treatment option	IA
According to the EMA label, pembrolizumab or atezolizumab could be an option in cisplatin ineligible patients with high PD-L1 expression levels	IIIB
Immune checkpoint inhibitors and chemotherapy as maintenance or second-line	
Maintenance therapy with avelumab is the standard of care for patients whose disease respond or did not progress after four to six cycles of first-line platinum-based chemotherapy (CG or CaG)	IA
After progression to a first-line platinum-based therapy, PD-1/PD-L1 inhibitors are standard options	
Pembrolizumab	IA
Atezolizumab	IIIB
Treatment with vinflunine is an alternative for patients in whom anti PD-1/PD-L1 therapy is not possible	IIB
Treatment after failure to chemotherapy and immune checkpoint inhibitors	
For patients progressing after platinum-containing chemotherapy and CPI, enfortumab-vedotin is recommended as standard treatment	IA
For patients progressing after platinum-containing chemotherapy with or without previous CPI, with tumor harboring FGFR mutations or fusions, erdafitinib could be considered	IIIB
Early supportive care is strongly recommended	VA

*LE* level of evidence; *GoR* grade of recommendation. *RC* radical cystectomy. *5FU* 5-fluorouracil. *LND* lymphadenectomy. *MIBC* muscle-invasive bladder cancer. *TMT* Trimodal therapy. *TURBT* transurethral resection of bladder tumor. *CG* Cisplatin-Gemcitabin. *CaG* carboplatin-Gemcitabine. *PD-1/PD-L1* Programmed Death-1/ Programmed Death-ligand 1. *CPI* check-point inhibitors. *FGFR* fibroblast growth factor receptor

.

In the metastatic setting, histologic classification may guide treatment selection. Platinum-etoposide combinations are recommended in SCBC, where treatment is often extrapolated from small cell lung cancer. Bladder adenocarcinomas and urachal carcinomas share molecular similarities with colorectal cancer, and based on multiple retrospective case reports, combination chemotherapy with a 5-FU based regimen such as FOLFOX6, with or without bevacizumab, should be considered [67].

There is growing evidence suggesting CPIs have a role in the treatment of variant and non-urothelial BC, mainly in the platinum-refractory setting. Overall, different series have demonstrated comparable ORR, PFS and OS between pure and other histologic subtypes treated with CPIs, although those with neuroendocrine features had worse outcomes [68].

There is no efficacy data on variant BC treated with EV. However, expression of Nectin-4 seems to be high (60–70%) in micropapillary tumors, nested carcinomas and plasmacytoid tumors, while extremely low or absent in sarcomatoid and small cell carcinomas, suggesting this drug may play a role in selected histologic subtypes [69].
